# Comparison of the scientific performance in hip and knee arthroplasty between the leading continents

**DOI:** 10.3389/fsurg.2023.1223905

**Published:** 2023-11-17

**Authors:** Milan Anton Wolf, Philipp Winter, Stefan Landgraeber, Patrick Orth

**Affiliations:** Department of Orthopaedic Surgery, Saarland University, Homburg, Germany

**Keywords:** hip, knee, arthroplasty, comparative study, continents, research

## Abstract

**Background:**

Scientific progress in the field of knee and hip arthroplasty has enabled the preservation of mobility and quality of life in the case of patients with many primary degenerative and (post-) traumatic joint diseases. This comparative study aims to investigate differences in scientific performance between the leading continents in the field of hip and knee arthroplasty.

**Methods:**

Using specific search terms all studies published by the scientific leading continents Europe, North America, Asia and Oceania listed in the Web of Science databases were included. All identified publications were analysed and comparative conclusions were drawn regarding the qualitative and quantitative scientific merit of each continent.

**Results:**

Europe, followed by North America, Asia, and Oceania, had the highest overall number of publications in the field of arthroplasty. Since 2000, there has been a strong increase in knee arthroplasty publication rate, particular pronounced in Asia. Studies performed and published in North America and those on knee arthroplasty received the highest number of fundings. Publications regarding hip arthroplasty achieved the highest average citation rate. In contradistinction to the others, in North America most funding was provided by private agencies.

**Conclusion:**

Although Europe showed the highest total number of publications, authors and institutions, arthroplasty research from North America received greater scientific attention and financial support. Measured by citations, publications on hip arthroplasty attained higher scientific interest and studies on knee arthroplasty received higher economic affection.

## Background

Scientific progress in the field of knee and hip arthroplasty has enabled the preservation of mobility and quality of life in the case of patients with primary degenerative and (post-) traumatic joint diseases. Due to increasing life expectancy, not only the number of necessary surgical interventions (1) but also the costs for the health care system are rising drastically (2). To cope with the demand, constant further development of implants and surgical techniques is essential. In recent years, the growing number of scientific achievements and publications in the field of hip and knee arthroplasty has enabled significant improvements in patient care (3).

Comparative analyses evaluate the scientific development in certain fields of research in accordance with scientific standards. This involves recording all publications in a thematic field (4). Subsequently, by analysing the baseline data, conclusions are drawn about the quantity of published research papers (number of publications, authors, institutes, journals, etc.). Further analyses allow conclusions on the quality of the research (impact factor of the journals, citation rate, source of funding, h-index). This enables scientifically substantiated statements and comparisons to be made about the individual areas of research.

In addition to differences in the quantity of publications on individual fields of hip and knee arthroplasty, there are also major differences in the publication behaviour of the countries and continents involved (5–7). Even though the quantitative increase in the field of hip and knee arthroplasty has already been investigated (8), it is still unclear how the qualitative variances unfold. The studies of hip and knee arthroplasty performed to date (9, 10) do not allow comparison due to methodological differences. Identifying the key differences between the arthroplasty subgroups may have a catalysing effect on still underrepresented research areas and regions in the future if the respective gaps are closed. Elucidating how publication behaviour varies in the individual continents should create a scientific equality of opportunity which could lead to a global improvement in patient care through an increase in high-quality scientific work.

Currently, the dynamics of publication in hip and knee arthroplasty across the leading continents remain unknown. Comparative analyses can be of assistance, as they aim to structure the scientific contributions to a topic and show in which countries, at which institutions, and in which disciplines the most research has been conducted. Therefore, this study aims at answering the following questions regarding such intercontinental differences: 1. Are there significant differences in the mere publication output in hip and knee arthroplasty? 2. Are there qualitative differences in the publication behaviour (e.g., citations and h-index)? 3. Are there differences in funding?.

## Methods and material

### Study design

**Figure 1:**
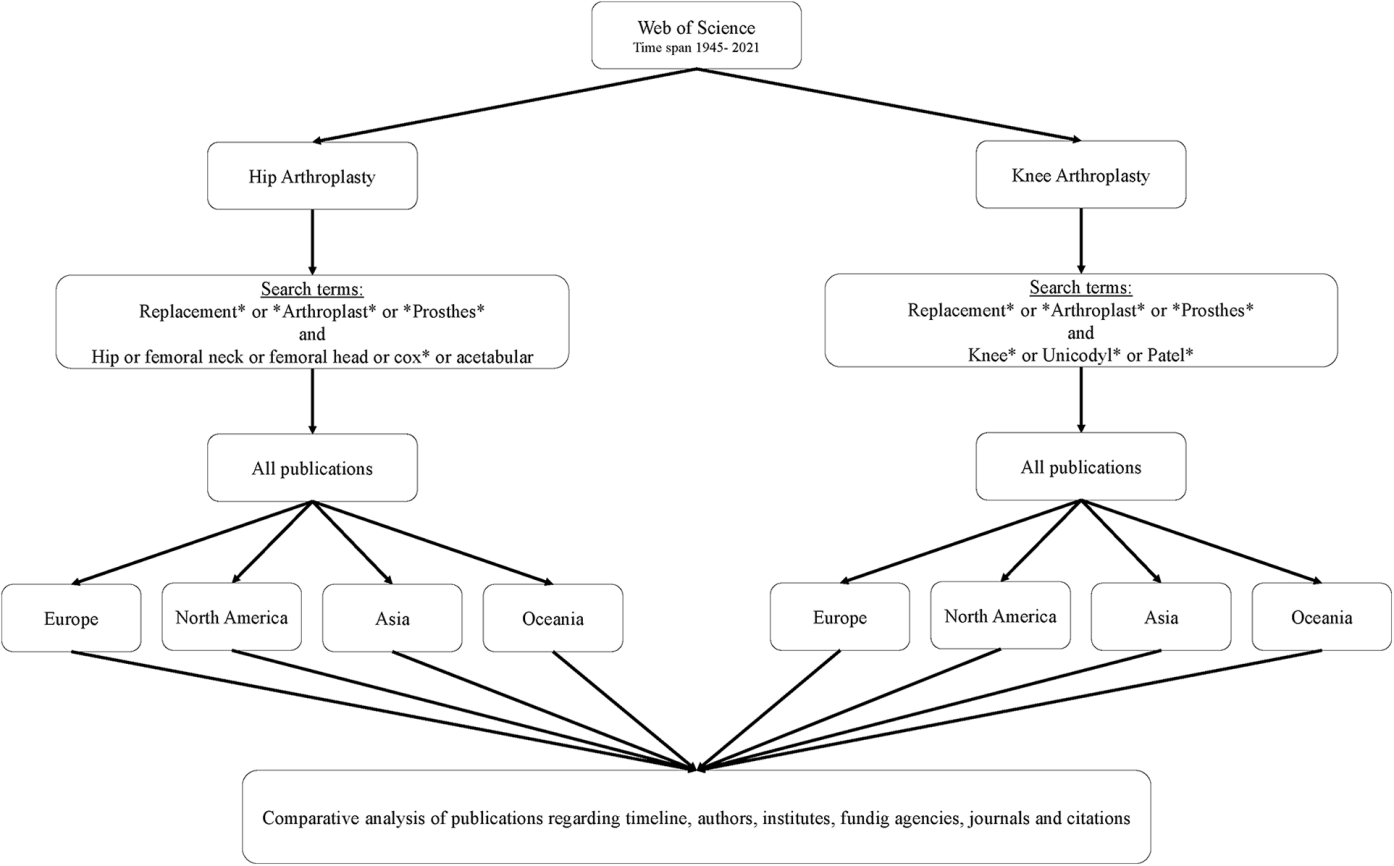
Study design, search terms data extraction and analysis.

In this study, publications on the clinically most relevant subgroups of arthroplasty for hip and knee joints were investigated. All publications from 1945 to 2021 (date of data collection: 28 August 2022) were recorded, analysed, and compared in accordance with the principles of infometrics. Here, regional differences were worked out on a continental level, whereby, for reasons of representativity, only the four continents with the most publications—Europe (EU), North America (NA), Asia (AS) and Oceania (OC)—were included (11, 12) (Figure 1).

### Database and search strategy

Data collection was carried out using the worldwide established, multi-disciplinary search platform for bibliographic databases Web of Science™ (WoS) (13, 14). To ensure high quality, specificity, and comparability of the results, the search only covered the title of publications. To include as many publications as possible on the topic of arthroplasty and at the same time prevent the inclusion of non-topic-related publications, broad but specific search terms and matching boolean operators were identified in accordance with the WoS guidelines. To generate a data set that is as representative as possible, the most common search terms were combined (Figure 1). The search terms were chosen based on their clinical and scientific importance and on previous bibliometric work in this area. Random verifications of the generated findings were carried out. Due to the vast number of publications, completing a full manual review was not possible. Different types of publications (e.g., article, review article and meeting abstracts) have been integrated in the analysis.

### Data extraction

The four continents with the largest publishing power (15) were selected and compared. Countries that are located in two continents were assigned to the continent in which the largest geographic proportion of the country's area is located. The analysis of the countries and thus the final assignment to a continent was based on the location of the first author's institute.

The publications were subsumed under the term arthroplasty and compared within the subgroups hip and knee arthroplasty between the continents. Here, the time course and the general publication rate were collected by the WoS analysis function and exported into an Excel table (Microsoft Corporation, Redmond, Washington, USA) for further statistical evaluation.

The authors and institutions were collected by the WoS analysis function and exported to an Excel table for further statistical calculation.

### Analysis

The compound annual growth rate (CAGR = ${\left(\frac{ending\;publications}{beginning\;publications}\right)}^{\left(\frac{1}{number\;of\;years}\right)}-1$) was calculated in order to enable a better comparison of the temporal course.

The funding agencies and publication titles were assigned to the corresponding continents by the WoS analysis function. Due to the large amount of data and the necessity of a manual analysis only, the top 100 agencies and publication titles were identified by means of their number of fundings or publication volume. The agencies were then assigned to either the private sector (e.g., companies) or the non-private sector [non-profit, governmental, and non-governmental organisations (NGOs)] according to their economic background. The journals were manually ranked regarding their current impact factor (IF) of the Journal Citation Report (https://jcr.clarivate.com). For journals that are no longer published, the IF of the last year of publication was used. If the journal had been renamed in the meantime, the IF of the current journal was used. The geographical assignment was made according to the information in the Journal Citation Report.

The citations were evaluated by the WoS Citation report. As for topics with more than 10,000 publications a corresponding analysis with the WoS analysis function is technically not feasible, therefore a cumulative citation analysis with two separate analyses of the publications from 1945 to 2014 and from 2015 to 2021 was performed. The results were then summarized and further processed.

Significance was assumed at *p* < 0.05. For statistical evaluation and creation of the figures GraphPad Prism 9 (Graphpad Software, Inc, San Diego, CA) and Microsoft Excel table were applied.

## Results

### Publication timeline

The largest number of publications on the topic of hip and knee arthroplasty derived from European researchers, followed by those from North America, Asia, and Oceania, (EU: hip: 12,817, knee: 10,031; NA: hip:8,887, knee: 9,383; AS: hip: 5,054, knee: 6,298, OC: hip: 2,031, knee: 1,626). Europe and Oceania had more publications in the field of hip arthroplasty than in knee arthroplasty. The opposite was true for North America and Asia.

**Figure 2:**
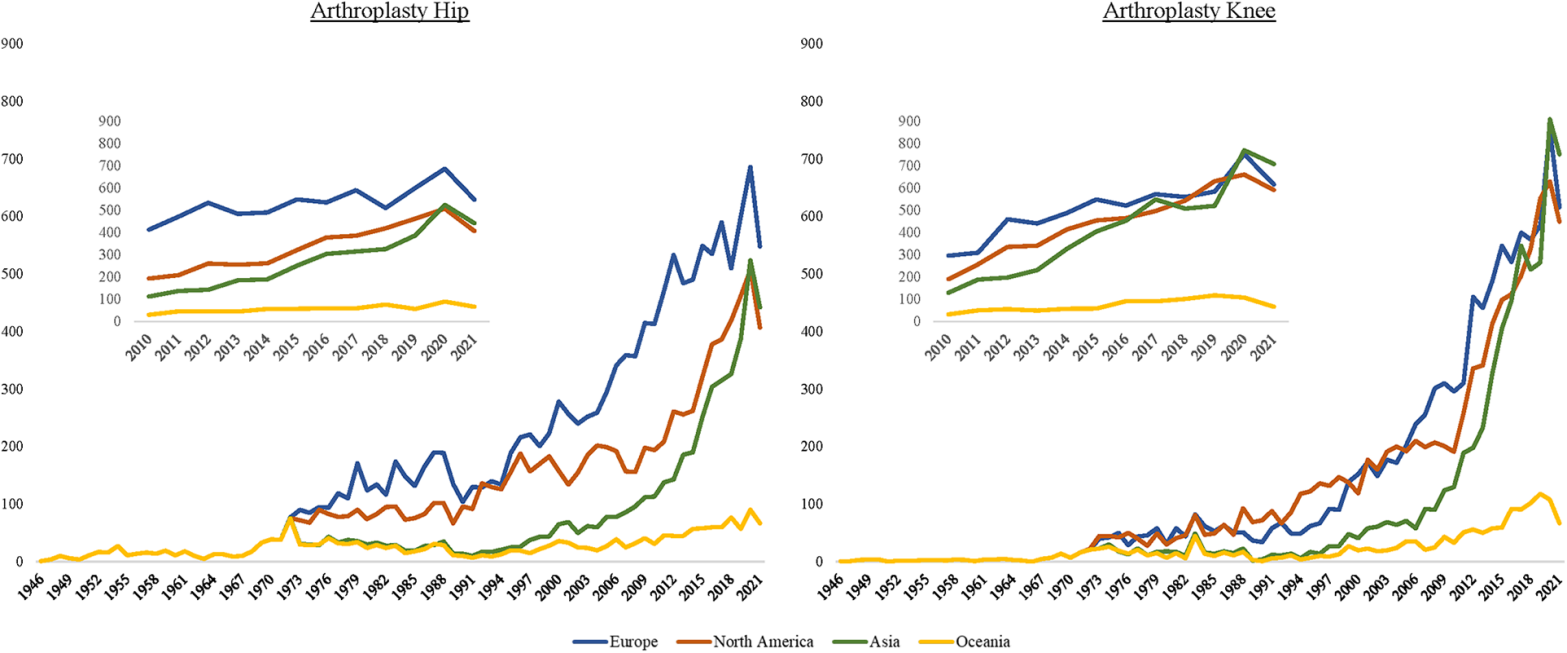
Time course of publications on hip arthroplasty (left graph) and knee arthroplasty (right graph). The inlet depicts the progress since the year 2010.

Europe consequently also achieved the highest compound annual growth rate (CAGR) of publications in the period under consideration (CAGR since 1945: EU: 9.46%, NA: 9.14%, Asia: 9.14%, OC: 6.41%). Since 2000, there has been a relative decrease in CAGR in Europe and North America. In contrast, in the two other continents, and Asia in particular, there has been an increase in the publication rate. The overall decrease is mainly due to the reduced publication performance in the field of hip arthroplasty (CAGR since 2000: EU: total: 6.96% hip: 4.92%; NA: total: 8.01%, hip: 7.05%; AS: total: 13.32%, hip: 11.00%; OC: total: 6.55%, hip: 4.63%) (Figure 2). In the research field of knee arthroplasty, there has been a proportionally greater surge in publications in recent years(CAGR since 2000 in knee arthroplasty: EU: 9.59%, NA: 9.07%, AS: 15.79%, OC: 8.90%).

### Authors

Europe showed the highest number of authors contributing to publications on hip and knee arthroplasty in absolute terms (EU: 50,620, NA: 35,474, Asia: 23,623, OC: 7,781; authors/publication: EU: hip: 2.14, knee: 2.24; NA: hip: 2.05, knee: 1.84; AS: hip: 2.15, knee: 2.02; OC: hip: 2.09, knee: 2.18).The average number of four authors per publication in the field of arthroplasty was not statistically significant. (number of authors per publication: EU: hip: 4.73, knee: 4.95; NA: hip: 4.65, knee: 4.41; AS: hip: 4.44, knee: 4.83; OC: hip: 3.17, knee: 3.60; *p* = .758).

### Institutes

In opposite to Asia and Oceania, more institutes were involved in the field of hip arthroplasty in Europe and North America (EU: hip: 6,269, knee: 5,914; NA: hip: 3,732, knee: 2,784; AS: 1,899, knee: 2,519; OC: hip 346, knee: 412). Europe and North America each had the highest average number of institutes per single publication (institutes involved per publication: EU: hip: 2.56, knee: 2.74; NA: hip: 2.74, knee: 2.34; AS: hip: 1.32, knee: 1.63; OC: hip: 0.80, knee: 1.29). The institutes with the most publications for each subgroup were located in North America (Table 1).

**Table 1:** Representation of the institutes with the most publications in the subject areas hip and knee arthroplasty.

Hip arthroplasty		Knee arthroplasty	
Institutes	Publications	Institutes	Publications
Harvard University	651	Mayo Clinic	559
Mayo Clinic	574	Hospital of Special Surgery	544
Hospital of Special Surgery	573	Harvard University	540
University of California System	492	University of California System	396
University of Toronto	441	University of Oxford	408

### Funding agencies

The largest number of agencies active in research funding was found in Europe. Except for Europe, most funding agencies were involved in knee replacement research (EU: hip: 2,156, knee: 2,068; NA: hip: 1,494, knee: 1,590, AS: hip: 774, knee: 961, OC: hip: 218, knee: 282).

More significantly, however, the most funding in absolute and thus also relative terms took place in North America (funded publications/fundings per publication: EU: hip: 4,993/0.39, knee: 4,750/0.46; NA: hip:4,647/0.52, knee: 5,422/0.58; AS: hip: 1,481/0.29, knee: 1,940/0.30; OC: hip: 424/0.21, knee: 505/0.31). This also shows that, except for Europe, publications on the subject of knee arthroplasty received more frequent funding.

**Figure 3:**
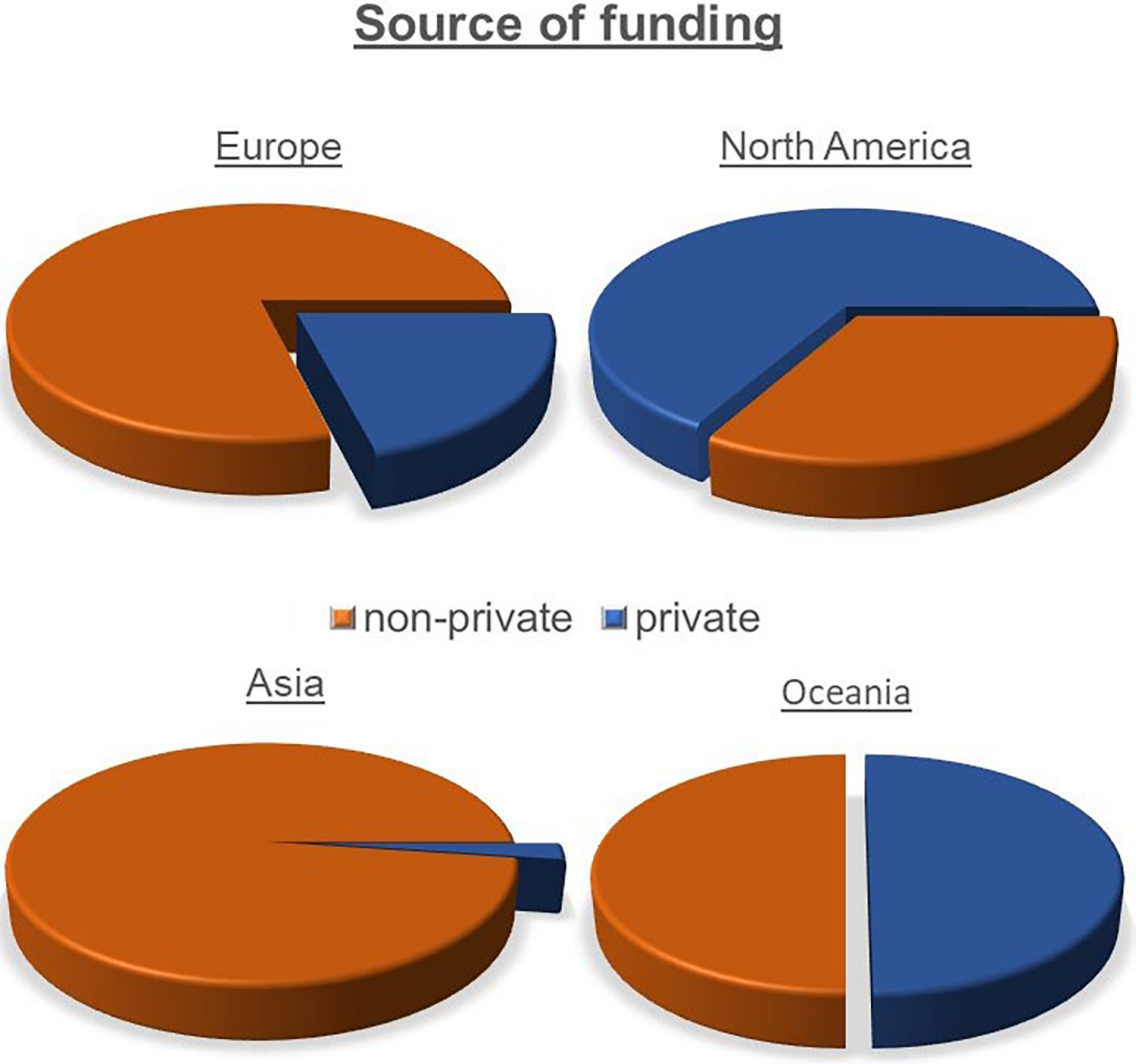
Pie chart showing the economic background of the funding agencies of each continent. The orange portion represents non-private [governmental, non-profit organisations and non-governmental organisations (NGOs)] agencies. The blue portion represents private agencies.

In Asia, funding was almost exclusively provided by non-private agencies (private: 2.2% vs. non-private: 97.8%). In Europe, the largest amount of funding was provided by non-private agencies too (private: 21.1% vs. non-private: 79.0%), while in Oceania, the ratio was balanced (private: 50.0% vs. non-private: 50.0%). In North America, on the other hand, funding was mainly provided by private agencies (private: 66.7% vs. non-private: 33.3%) (Figure 3).

### Citations

Publications from North America received the most citations per publication in both subject areas. Publications on the field of hip arthroplasty achieved on average more citations than publications on knee arthroplasty (average citations per publication; EU: hip: 20.77, knee: 20.29; NA: hip: 32.57, knee: 27.62, AS: hip: 10.86, knee: 10.22, OC: hip: 14.36, knee: 12.32) (Table 2).

**Table 2:** Presentation of the citation metrics.

Hip arthroplasty	Europe	North America	Asia	Oceania
Citing articles	100,037	92,773	30,974	21,619
Times cited	266,248	289,449	54,871	29,172
Average citation per publication	20.77	32.57	10.86	14.36
Knee arthroplasty				
Citing articles	66,707	77,216	28,593	14,750
Times cited	203,528	259,158	64,374	20,034
Average citation per publication	20.29	27.62	10.22	12.32

### Publication titles/journals

European studies were published in the largest number of different journals (journals: EU: hip: 1,178, knee: 887; NA: hip: 745, knee: 698; AS: hip: 775, knee: 758; OC: hip: 218, knee: 291). Regarding the 100 most popular journals, Europe, Asia and Oceania showed a preference for publishing in European journals (publications from EU: 78% EU journal, 22% NA journal; publications from NA: 32% EU journal, 61% NA journal, 4% AS journal; publications from AS: 51% EU journal, 24% NA journal, 24% AS journal, 1% OC journal; publications from OC: 47% EU journal, 37% NA journal, 2% AS journal, 8% OC journal). Publications from Oceania among the 100 most popular journals achieved the highest average impact factor (IF: EU: 3.76, NA: 4.80, AS; 3.89, OC: 5.52).

## Discussion

In this study, we were able to show for the first time that the highest number of publications in the field of hip and knee arthroplasty between 1945 and 2021 derived from Europe, followed by North America, Asia and Oceania. In the last 20 years, Asia has shown a particularly strong increase in publication rate. Most authors and participating institutes were located in Europe. Publications regarding knee arthroplasty received the most funding, whereas publications about hip arthroplasty achieved more citations. Funding in Asia tended to involve almost exclusively non-private agencies. In North America, on the other hand, most funding was provided by private agencies. Researchers from Europe, Asia and Oceania published mainly in European journals, while North American researchers published mostly in journals from North America. Interestingly, publications from Oceania, yielded the highest cumulative IF.

In the field of joint replacement, there have been significant innovations and improvements since the first studies on this topic. Evidence of this is provided by the growing number of published research papers. In addition to the increasing quantity of publications observed in this study and others (3), there has also been an increase in the scientific field of orthopaedics (16) and of medicine in general (17). The rising number of arthroplastic interventions performed overall, and particularly in countries with a high gross domestic product (GDP), is a possible explanation for the fact that high-income countries such as the United States, Germany and England have a particular need for scientific progress and that this in turn leads to the highest publication rate (18–20). Only through scientific progress and the resulting improvement in prosthesis durability and revision techniques can patient care be guaranteed in the long term as expectancy and quality of life continue to rise (21). Although in absolute terms most publications dealt with the topic of hip arthroplasty, there has been a rapid increase in the rate of knee arthroplasty publications in recent years, particularly by Asian authors. This is probably due to the relatively greater number of patients requiring primary and revision knee surgery (1, 22), an increased demand for knee mobility (23) and a higher prevalence of osteoarthritis of the knee in the Asian region (24).

In this research, an average of four authors per publication was observed, with no significant variation across continents. This high average aligns with findings from other bibliometric studies (25) and is likely due to the mounting publication demands in the medical.

Regarding financial support, no statements could be made about the total amount of funding received (in the sense of value), as this analysis only refers to the number of funding grants awarded. Publications from North America received the most funding, predominantly by private funding agencies. As a proportional relationship exists between the financial support of an author, the average h-index and the number of publications (26, 27), this could be an explanation for the high scientific impact of North America. The notable differences in the origin of financial support (private vs. non-private)—which are particularly pronounced between North America and Asia—could possibly be due to the respective political attitudes of the main regional actors, i.e., The United States of America and the Republic of China.

As a probable consequence of the above-mentioned correlation between funding and scientific impact (28, 29) researchers from North America also achieved also the most citations for both subfields. In addition to the preferential financial support, there is also the possibility that North American authors prioritize citing publications from the United States (26).

Due to the high total number of journals identified in this study, a focussed analysis of the 100 journals with the most publications was carried out. This revealed the trend that North American authors prefer to publish in North American journals. Previous studies have already shown that the rate of paper acceptance in American journals differs significantly depending on the origin of the submitting author (24). Hence, regardless of the quality of the research study, the acceptance rates in American journals are almost twice as high for American authors (29, 30).

To ensure equal opportunities for scientific development in the future, the continents and their affiliated institutions must cooperate closely. All researchers must have access to the divergent scientific structures and expertise, which focus on sub-topics unique to each continent, to facilitate collaboration. In doing so, complementary strengths can be cultivated and weaknesses resolved.

### Limitations

Like all comparative studies, this study is subject to some limitations. Unspecific search terms may result in off-topic publications, while over-specific combinations can lead to the exclusion of relevant publications. Even though the Web of Science databases are among the most comprehensive databases, not all publications are represented and there are deficits especially with regard to non-English publications (13). The extensive number of publications makes manual screening of publications unfeasible, which can lead to the risk of including off-topic studies in the analysis or double-counting studies. Therefore, the additional use of data from multiple databases in one study would be advantageous but requires the support of an external program. Affiliations to nations are determined by the nationality of the first author, possibly reducing multicentre studies to this one nation. In a citation-based analysis, more recent publications have a lower chance of recitation when compared with older publications, irrespective of their impact. This should be taken into consideration when interpreting the number of citations (31–34).

## Conclusion

Although Europe had the highest total number of publications, authors and institutions, publications from North America received greater scientific attention and financial support. As measured by citations, publications on hip arthroplasty attained higher scientific interest and studies on knee arthroplasty received higher economic attention. In recent years, Asia has shown a particularly strong increase in publication rate.

## Data Availability

The original contributions presented in the study are included in the article/Supplementary Material, further inquiries can be directed to the corresponding author.
